# Supplementary material for: Comparison of LC-MS3 and LC-MRM Method for Quantifying Voriconazole and Its Application in Therapeutic Drug Monitoring of Human Plasma
Source: Molecules. 2022 Aug 31;27(17):5609. doi: 10.3390/molecules27175609 (PMC9457787; doi:10.3390/molecules27175609)
Supplement: Supplementary file 1 [file molecules-27-05609-s001.zip › molecules-1842328-supplementary.pdf]

supplementary materials

Comparison of LC–MS^3 and LC–MRM method for quantifying Voriconazole and its application in therapeutic drug monitoring of human plasma

2.4 Method Validation

Specificity is evaluated by analyzing plasma samples from six healthy volunteers. Selectivity is also investigated by spiking blank sample with IS and blank sample with LOQ to check the signals in the channel of the voriconazole. The linearity is examined using calibration standards in plasma samples at concentration ranging from 0.25 to 20 μg/mL on three non-consecutive days. An acceptable determination coefficient ((R^2=0.9996) should be attained. The LOQ is defined as the lowest concentration of the calibration curves. The reproducibility at LOQ level is evaluated by consecutively injecting six processed samples at LOQ level and comparing its precision and accuracy against the established linearity. A maximum of ≤20% variation is accepted.

Extraction recovery and matrix effect of analyte and IS are determined by analyzing six samples at three different levels (0.5, 2.5 and 10.0μg/mL). In this validation, three separate sets sample are prepared. Set A was made by spiking QC samples and IS in to blank plasma samples. Set B is prepared by adding voriconazole and IS at the same concentrations after the plasma extraction process (post-extraction addition). Set C is acquired by adding QC working solutions and IS into water. Recovery (%) = A /B *100, and Matrix effect (%) = B/C *100.

Precision (relative standard deviation, RSD) and accuracy (relative error, RE) are assayed by analyzing six sets of replicates of LOQ and QC samples. The intra-assay

precision and accuracy are evaluated by analyzing these samples on the same day, while inter-assay precision and accuracy are evaluated over three different days. The precision should not exceed 15% (20% for LOQ) of RSD and accuracy should be within 85–115% (80–120% for LOQ) of the nominal concentration.

The stability is evaluated for voriconazole in human plasma by analyzing QC samples with 0.5, 2.5 and 10 μg/mL at different storage conditions. Long term stability is evaluated for plasma samples after storage at −80 ◦C for 2 weeks. Short term stability was studied on analysis of QC samples after three freeze-thaw, and at room temperature (RT) for 8 h. Processed samples stored at 8 °C for 6 h are reanalyzed for auto sampler stability. Under all conditions, a maximum loss of 15% of voriconazole concentration was acceptable stability.

Table S1. Concentrations of voriconazole in 42 human plasma samples are analyzed by HPLC-MS^2 and HPLC-MS^3 methods

Sample ID	HPLC-MS^2 (μg/mL)	HPLC-MS^3 (μg/mL)	(HPLC-MS^2/HPLC-MS^3)%
sample1	3.68	3.41	92.66
sample2	1.05	1.20	114.29
sample3	2.02	2.08	102.97
sample4	0.72	0.65	90.72
sample5	2.86	2.37	82.87
sample6	1.97	2.01	102.03
sample7	2.55	2.16	84.71
sample8	3.58	2.92	81.56
sample9	0.90	0.89	98.23
sample10	1.51	1.67	110.60
sample11	1.05	0.91	86.57
sample12	3.13	2.64	84.35
sample13	1.71	1.76	102.92
sample14	2.32	2.68	115.52
sample15	4.52	3.75	82.96
sample16	1.1	1.09	99.09

sample17	1.77	1.74	98.31
sample18	0.84	0.77	109.09
sample19	2.82	3.16	112.06
sample20	1.84	1.78	96.74
sample21	2.32	2.39	103.02
sample22	3.55	3.98	112.11
sample23	1.03	1.05	101.94
sample24	1.97	2.02	102.54
sample25	1.16	0.92	83.64
sample26	2.85	2.86	102.14
sample27	2.09	1.97	94.26
sample28	2.15	2.55	118.60
sample29	3.32	3.58	107.83
sample30	0.93	0.90	97.84
sample31	2.25	2.51	111.56
sample32	0.91	1.10	116.54
Sample33	3.53	3.13	88.67
Sample34	2.55	2.71	106.27
Sample35	2.39	2.32	97.07
sample36	5.63	5.52	98.05
sample37	1.28	1.10	85.94
sample38	2.40	2.77	115.42
sample39	0.98	1.14	116.33
Sample40	2.58	2.82	109.30
Sample41	2.07	1.84	88.89
Sample42	2.46	2.32	94.31
